# Supplementary material for: Depletion of Ly6G-Expressing Neutrophilic Cells Leads to Altered Peripheral T-Cell Homeostasis and Thymic Development in Neonatal Mice
Source: Int J Mol Sci. 2023 Apr 24;24(9):7763. doi: 10.3390/ijms24097763 (PMC10178674; doi:10.3390/ijms24097763)
Supplement: Supplementary file 1 [file ijms-24-07763-s001.zip › ijms-2331630-supplementary.pdf]

Supplementary Figure 1

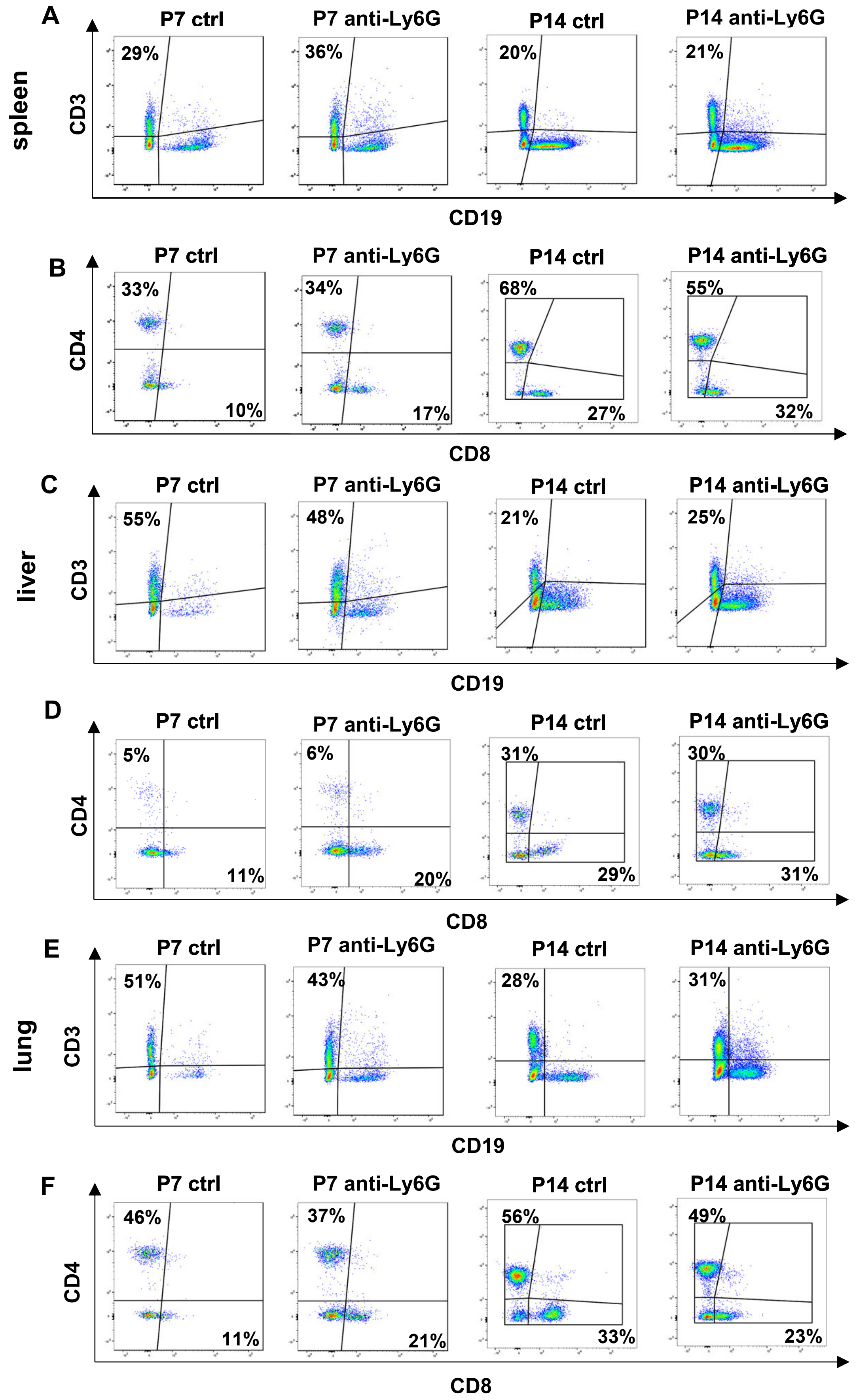

Supplementary Figure S1: Peripheral T-cell subsets after neutrophil depletion in neonatal mice. C57BL/6J mice were terminally mated. Two days before the expected litter date, the dam received an intravenous (i.v.) injection of a depleting anti-Ly6G antibody or an isotype control antibody into the tail vein. Injections were repeated every three days until the offspring were sacrificed. Offspring at the seventh postnatal day (P7) and at the 14th postnatal day (P14) were euthanized and organs were collected. Tissue was homogenized and filtered to obtain single cell suspensions. Cells were then analyzed by flow cytometry. (A-F) Density plots for CD19 vs. CD3 (A, C, E) and CD8 vs. CD4 showing the gating strategy for total T-cells and $CD4^+$ and $CD8^+$ T-cells.

Supplementary Figure S2

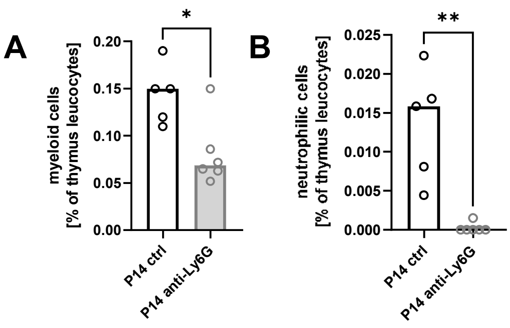

Supplementary Figure S2: Depletion of thymic neutrophils by antibody application to the dam. C57BL/6J mice were terminally mated. Two days before the expected litter date, the dam received an intravenous (i.v.) injection of a depleting anti-Ly6G antibody or an isotype control antibody into the tail vein. Injections were repeated every three days until the offspring were sacrificed. Offspring at the 14th postnatal day (P14) were euthanized and thymi were collected. Tissue was homogenized and filtered to obtain single cell suspensions. Cells were then analyzed by flow cytometry. (A+B) Scatter diagrams with bars showing percentages of myeloid cells (A) and neutrophilic cells (B) from $CD45^+$ leucocytes in thymi of newborn mice without (white bars) and with (grey bars) neutrophil depletion at P14. Each symbol represents an individual sample and the median is indicated; n=5-6, *p<0.05, **p<0.01; Mann Whitney test.

Supplementary Figure S3

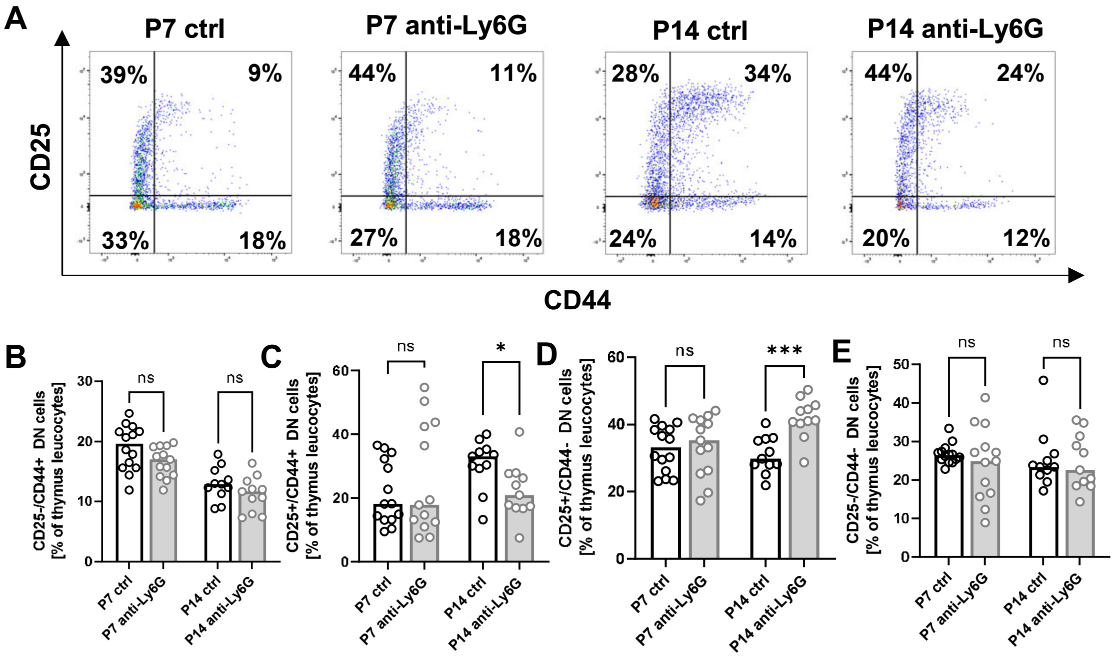

Supplementary Figure S3: Double negative subsets in thymocytes after neutrophil depletion. C57BL/6J mice were terminally mated. Two days before the expected litter date, the dam received an intravenous (i.v.) injection of a depleting anti-Ly6G antibody or an isotype control antibody into the tail vein. Injections were repeated every three days until the offspring were sacrificed. Offspring at the seventh postnatal day (P7) and at the 14th postnatal day (P14) were euthanized and thymi were collected. Tissue was homogenized and filtered to obtain single cell suspensions. Cells were then analyzed by flow cytometry. (A) Density plots for CD44 vs. CD25 showing the populations of double negative (DN) subsets DN1-DN4 in thymi of newborn WT mice without or with neutrophil depletion at P7 (left) and P14 (right). Cells were pre-gated on living lineage negative and $CD4^-/CD8^-$ cells. (B- E) Scatter diagrams with bars showing percentages of DN1 (B), DN2 (C), DN3 (D) and DN4 (E) thymocytes in thymi of newborn mice without (white bars) and with (grey bars) neutrophil depletion at P7 and P14. Each symbol represents an individual sample and the median is indicated; n=11-14, *p<0.05; ***p<0.001;; ns not significant; Mann Whitney test.

Supplementary Figure S4

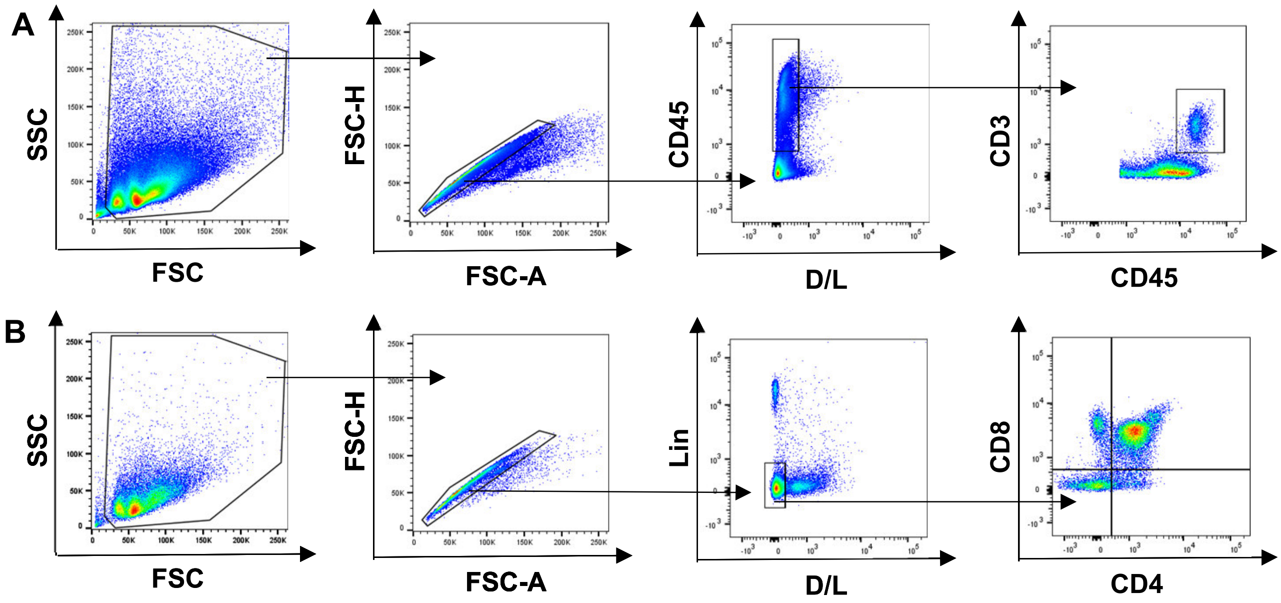

Supplementary Figure S4: Gating strategies for splenocytes and thymocytes.
(A+B) Density plots showing the gating strategy for splenocytes (A) and thymocytes (B).
